# Intense exercise training is not effective to restore the endothelial NO-dependent relaxation in STZ-diabetic rat aorta

**DOI:** 10.1186/1475-2840-12-32

**Published:** 2013-02-11

**Authors:** Mohamed Sami Zguira, Sophie Vincent, Solène Le Douairon Lahaye, Ludivine Malarde, Zouhair Tabka, Bernard Saïag

**Affiliations:** 1 Laboratory “Movement Sport and health Sciences”, UFR APS University of Rennes 2, Avenue Charles Tillon, Rennes cedex 35044, France; 2 Laboratory “Movement Sport and health Sciences”, ENS Cachan –antenne de Bretagne, Campus Ker Lann, Bruz, France; 3 Clinical Laboratory of Physiology, Medical School of Sousse, Sousse, Tunisia

**Keywords:** Endothelial relaxation, Training, Acetylcholine, ADPβS, Diabetic rat

## Abstract

**Background:**

The aim of this study was to examine the effects of intense physical training on vascular function in streptozotocin-diabetic rats. We focused on the endothelium-dependent relaxation (EDR) induced by acetylcholine (ACh) and stable ADP adenosine-5′- O – (2-thiodiphosphate) (ADPβS).

**Methods:**

Control or diabetic male Wistar rats (n=44) were randomly assigned to sedentary or trained groups. The training program consisted in a regular period of running on a treadmill during 8 weeks (10° incline and up to 25 m/min, 60 min/day). The reactivity of isolated thoracic aorta rings of healthy, diabetic and/or trained has been tested.

**Results:**

ACh and ADPβS-induced EDR were observed in phenylephrine (PE) pre-contracted vessels. As compared to sedentary control group, diabetic rats showed an increase in PE-induced contraction and a decrease in ACh and ADPβS-induced EDR (p<0.05). Moreover, there were no increase in ACh and ADPβS-induced EDR in diabetic rats. N-Nitro-L-Arginine Methyl Ester inhibited the nitric oxide synthase in diabetic and control rats, thereby resulting in a strong inhibition of the EDR induced by ACh and ADPβS (10^-6^ M).

**Conclusion:**

Diabetes induced an endothelium dysfunction. Nevertheless, our intense physical training was not effective to restore the aorta endothelial function.

## Introduction

The endothelium provides a cellular lining to all blood vessels in the circulatory system and forms a structural barrier between the vascular space and the tissues. Endothelial cells (ECs) regulate vascular flow and basal vasomotor tone by the highly controlled release of vasodilators (nitric oxide (NO) and prostacyclin (PGI2)) and vasoconstrictors [1]. NO is generated in ECs by the oxidation of L-arginine to L-citrulline by the NO synthases (NOS) enzymes family. The endothelial NOS (eNOS) isoform is constitutively active but is further induced by receptor dependent agonist such as thrombin, adenosine 5-diphosphate, bradykinin and substance P [2,3]. Shear stress also stimulates eNOS activity by virtue of a shear response consensus sequence GAGACC in the eNOS gene promoter [4]. NO has pleiotropic effects on the vasculature. It causes vascular smooth muscle relaxation by binding to guanyl cyclase hence maintaining basal vasomotor tone. It also plays a critical role in the inhibition of thrombosis by inhibiting platelet adhesion activation and agonist-induced secretion. Moderate exercise training is well known to induce beneficial effects by improving Endothelial Function (EF)] and EDR in healthy subjects [5-8]. Efficiency of training depends on both duration and frequency of that type of exercise [2,9-11]. Training, carried out in variable intensities of exercise, allows authors to note that moderate intensities potentiate the endothelium-dependent-relaxation (EDR) [2].

Diabetes is a risk factor in the development of both macro- and microvascular diseases [12]. It has been established that diabetes is associated with vascular dysfunctions caused by an impairment of EDR [13,14]. Endothelial dysfunction (ED) is commonly considered as a biomarker of cardiovascular risk and the importance of the endothelium in maintaining normal vascular function is also well recognized [15,16]. In patients with type 1 or type 2 diabetes, the forearm blood–flow (dilator) responses to ACh are reduced, suggesting an ED [17-19]. In type 1 diabetic animal model, EDR induced by ACh is also impaired. The decrease in NO bioavailability is one of the reasons in this altered-relaxation [20,21]. Moderate physical activity has been reported to be a major component in the clinical management of diabetic patients [22]. However, the effects of intense exercise training on EF in healthy or pathological subjects are scarce and controversial [23]. Thus, the medical community generally advises diabetic patients to undertake physical activity of moderate intensity (40 to 60% $\dot{V}$ O_2max_) for 20 to 30 minutes at least 3 to 5 times per week. As vigorous physical activity can acutely and transiently increase the risk of cardiovascular events, many practitioners encourage diabetic patients to avoid intense and heavy exercise. However, many type 1 diabetic athletes are known to practice at a high level of competition and to win Olympic medals, leads us to believe that intense sports activity is not necessarily detrimental to type 1 diabetic patient. Moreover, recently, our laboratory showed that intense exercise training combined to insulin treatment had beneficial effects on basal cardiac function and adaptation in expression and responsiveness of cardiac receptors in type 1 diabetic rats [24,25]. Furthermore, high intensity training can be more effective than moderate intensity training to improve cardiovascular health and clinical outcome in patients with metabolic syndrome [26]. Other data showed that a high-intensity interval training model was effective for improving skeletal muscle mitochondrial capacity and functional exercise performance [27]. Finally, mechanism of the ACh-induced EDR is nowadays well known in diabetic or healthy subjects [28-30], on the contrary, stable ADP adenosine-5^′^- O – (2-thiodiphosphate) ADPβS-induced EDR mechanisms have been described so far only in healthy rats. The ADPβS-induced relaxation is endothelium-dependent, mediated by P2Y purinoreceptors, at least in part linked to NO and prostacyclin release, and eventually Endothelium-Dependent Hyperpolarizing Factor (EDHF) [31]. To our knowledge, the effects of moderate and intensive training on ADPβS-induced EDR on healthy and diabetic subjects or rats are not documented. This study aimed to evaluate the effects of intense exercise training on EDR in diabetic rats as well as the NO-EDR induced by ACh and ADPβS.

## Materials and methods

### Experimental model

Animal experiments were conducted according to the Guide for the Care and Use of Laboratory Animals. All experimental protocols were approved by the institutional Animal Care and Use Committee of Rennes University (France) and were carried out following the French Agricultural Ministry’s to the care and use of laboratory Animals. This study meets the ethical standards in sports and exercise science research of the international journal of sports medicine [32]. The study was conducted in male Wistar rats, housed in an animal room on an inverse 12:12-h light-dark cycle (temperature 21 ± 1°C) and given access to water and food ad libitum. The rats (n = 44; from Janvier, France), 9 week-old at the beginning of the experiment, were randomly divided into 4 groups: sedentary control (SC, n = 10), trained control (TC, n = 10), sedentary diabetic (SD, n = 14), and trained diabetic (TD, n = 10).

### Induction of experimental streptozotocin (STZ)–induced diabetes

This experimental model of rats made diabetic with streptozotocin (STZ) injection has been validated in previous studies [24]. Animals were injected with either an intraperitoneal single dose of STZ in 0.1 M citrate buffer, pH 4.5 (45 mg/kg) (SD, TD) or citrate buffer only (SC, TC). Three days later, blood glucose levels were determined using a glucometer (MediSense Optium). The onset of diabetes was determined by blood glucose concentration > 250 mg/dL. The detection of ketone bodies, using a glucometer or urinary strips (Keto-Diastix^®^-Bayer Diagnostic) and body weight loss was used to confirm diabetes.

### Exercise training program

One week after the STZ or citrate buffer injection, animals were exercised. Physical training consisted in a progressive running up to 25 m/min, 60 min, 5 days/week (for 8 weeks), as previously validated in our laboratory in same models [24,25] on a rodent treadmill (Exer 3/6 treadmill, Columbus Instruments) set at an incline of 10°. For the first 2 weeks, each exercise bout consisted of 10 min of running at 20 m/min. The following 3 weeks consisted progressively of 40 min at 22 m/min. for the remaining 3 weeks of the protocol; the duration was increased to 60 min at 25 m/min. Only animals which ran steadily on the treadmill were included in the study. All rats were sacrificed 24 h after the last training session.

### Anesthesia & sacrifice

24 to 48 h after the last session of exercise to avoid immediate effects of exercise [2], animals were anesthetized with sodium pentobarbital (50 mg/kg) administrated intraperitoneally. The thoracic aorta were gently removed and immediately stored in a Krebs-Henseleit solution oxygenated and maintained at 37°C (pH 7.4) for vascular analyses. The gastrocnemius tissue was removed and immediately frozen in liquid nitrogen for subsequent analyses.

### Citrate synthase activity

Frozen gastrocnemius tissue (200 mg) was used to assay citrate synthase (CS) activity. Muscle samples were homogenized (1/10 w/v) in a buffer solution pH 7.5 containing Na_2_HPO_4_ (0.1 mol.L^-1^), NaH_2_PO_4_, H_2_O (0.1 mol.L^-1^), and EDTA (2 mmol.L^-1^) for 20s at 30000 rpm with a polytron. The homogenate was then sonicated 6 x 10s and centrifuged at 1500 G for 13 min at 4°C in duplicate. Citrate synthase activity was spectrophotometrically determined in duplicate in protein extracts at 25°C. Results were expressed in μmol.ml^-1^.min^-1^.gTissue^-1^ for each group. [24,25].

### Preparation of aortic rings for vascular analyses

The removed-thoracic aorta were stored in a Krebs-Henseleit solution oxygenated and maintained at 37°C (pH 7.4) (composition in mM : Nacl 95, KCl 5, MgSO_4_ 1.2, KH_2_PO_4_ 1.2, NaHCO_3_ 24.9, CaCl_2_ 2.6, glucose 10) and bubbled with 95% O_2_-5% CO_2_. The surrounding connective tissue was carefully removed and rings (5 mm width) were prepared. Extreme care was taken not to stretch or damage the luminal surface of the aorta to ensure the integrity of the endothelium. The rings of the proximal portion of the aortic arch were mounted horizontally under isometric conditions in a 15 ml organ bath by inserting two stainless-steel wires into the lumen, according to the Bevan & Osher method (1972) [33]. The tissue isometric tension (g) was recorded by a force-displacement transducer (EMKA Technologies, Paris, France) and an acquisition system (IOx, EMKA Technologies, Paris, France). Preparations were allowed to reach equilibrium for at least 60 minutes under a resting tension of 600-900 mg. Before the beginning of each study, the contractile function was verified by obtaining a KCl-induced contraction and the functional state of the endothelium was evaluated by observation of relaxation to ACh on a ring precontracted with phenylephrine (10^-7^ M). Only the endothelial intact rings (more than 50% relaxation to Ach) were used.

After the washout (3 times for 20 min), the concentration-response relationship of PE was determined. The percentage of relaxation was evaluated in function of the contraction obtained with a 75% maximal effect of PE concentration. The relaxation study used ACh or ADPβS added in a cumulative manner with an increase of the concentrations (from 10^-6^ M to 10^-5^ M for ACh or from 10^-8^ M to 10^-7^ M for ADPβS). Finally, the preparations were washed 3 times before the addition of L NAME (10^-5^ M) until the reach of the basic tension observed (30 min). Then, the same protocol was applied with ACh and ADPβS under N-Nitro-L-Arginine Methyl Ester (L-NAME) conditions. In the L-NAME result section, we will show relaxation data in response to a single dose of ADPβS (10^-6^ M).

### Drugs used

The Acetylcholine (ACh), Phenylephrine (PE), N-Nitro-L-Arginine Methyl Ester (L-NAME) and streptozotocin (STZ) were obtained from Sigma (U.S.A). The Adenosine-5^′^- O – (2-thiodiphosphate) lithium salt (ADPβS) was from Boehringer Mannheim (Germany). All concentrations are expressed as final molar concentrations in the organ bath.

### Statistical analysis

Data are expressed as mean ± SEM; Animal characteristics and CS activity were compared using a 1 way ANOVA and the dose-response curves were compared performed by a 2-way ANOVA for repeated measures followed by Student-Newman-Keuls post-ANOVA tests (Statistica 7.1 software, StatSoft France, 2005). Values of p<0.05 were considered significant.

## Results

### Animal characteristics

Higher glycemia in SD rats than in SC rats (p<0.001) confirmed the diabetic state. Training reduced but not restored the levels of blood glucose, which were lower in TD rats compared to the SD (p<0.001) (Table 1). Body weights, at the end of the study, were significantly depressed in both SD and TD rats as compared to the SC rats (p<0.001). SC rats had significantly higher body weights than TC rats (p<0.05), whereas SD rats had significantly lower body weights than TD rats (p<0.05) (Table 1). All together, blood glucose values and body weight in diabetic group confirm their pathological status. The levels of gastrocnemius CS activity (mol.ml^-1^.min^-1^.gTissue^-1^) increased significantly (p<0.05) in trained rats compared to their respective sedentary counterparts (Table 1).

**Table 1:** General characteristics of animals

	**Sedentary Control (SC)**	**Trained control (TC)**	**Sedentary diabetic (SD)**	**Trained diabetic (TD)**
	n = 10	n = 10	n = 14	n = 10
Body weight (g)	504.1 ± 6.3	478.3 ± 10.3*	293.1 ± 11.4 * †	330.2 ± 11.4 * † ‡
Blood glucose (mg/dl)	128.7 ± 3.7	124.3 ± 3.7	556.2 ±13.6 * †	497.4 ± 23.8 * † ‡
Citrate synthase (μmol.mL^-1^.min^-1^.g tissue^-1^)	56 ± 1	69 ± 4*	61 ± 6	100 ± 9 ‡

Data are presented as mean ± SEM *= significantly different from sedentary control rats *(p<0.05),* † = significantly different from trained control rats, ‡ = significantly different from sedentary diabetic rats *(p<0.05).*

### Vasomotricity study

#### Phenylephrine (PE) Concentrations responses curves

**Figure 1:**
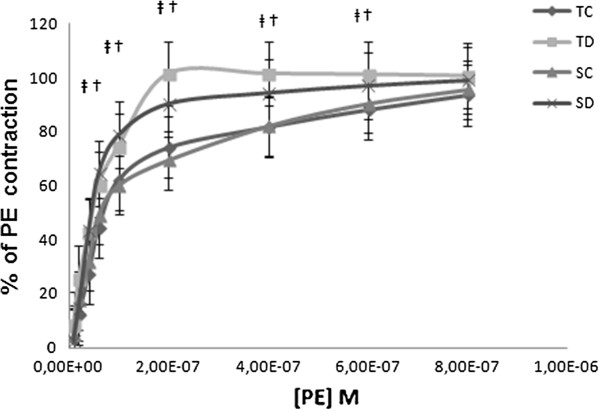
**Concentration dependent constriction of phenylephrine (%).** Concentration dependent contraction of phenylephrine (PE) on thoracic aorta rings obtained from sedentary control (SC), trained control (TC), sedentary diabetic (SD) and trained diabetic (TD) groups. Tension is expressed as % relaxation on initial contraction with PE. Values are expressed as mean ±SEM. ‡ Significantly different from sedentary diabetic rats (p<0.05). Significantly different from trained diabetic rats (p<0.05).

No significant differences were observed between groups TC and SC. TD and SD were significantly different for concentrations from 10^-7^ M to 6.10^-7^ M (Figure 1).

### Effect of diabetes on ACh and ADPβS-induced EDR

**Figure 2:**
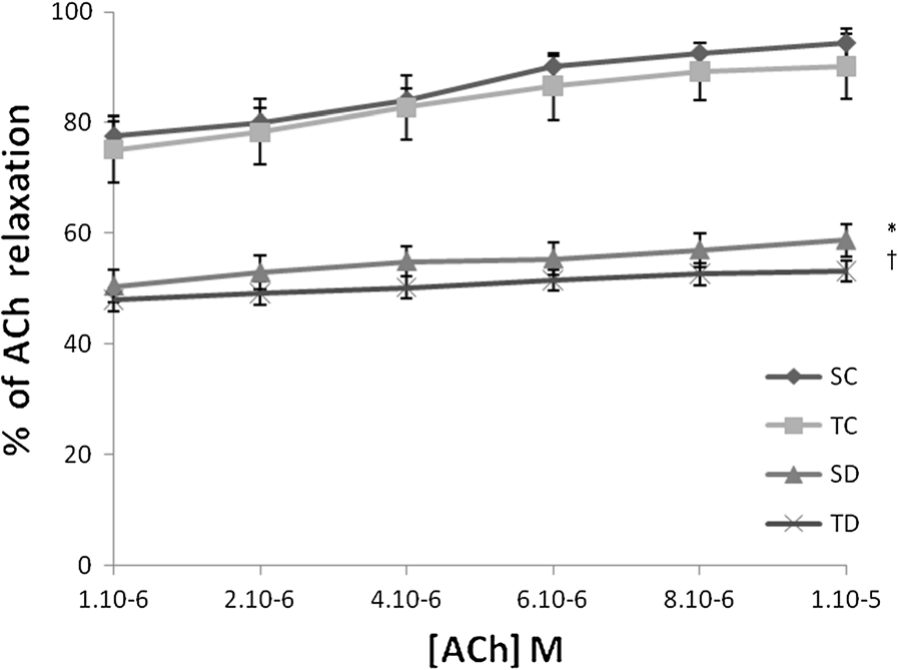
**Concentration dependent relaxation of acetylcholine (%).** Concentration dependent relaxation of acetylcholine (ACh) on thoracic aorta rings obtained from sedentary control (SC), trained control (TC), sedentary diabetic (SD) and trained diabetic (TD) groups precontracted with PE. Tension is expressed as % relaxation on initial contraction with PE. Values are expressed as mean ±SEM. * Significantly different from sedentary control rats (p<0.05), † significantly different from trained control rats.

We observe that the ACh as ADPβS induced relaxations on aortic rings in healthy and diabetic rats. Relaxations obtained from diabetic rats are significantly reduced compared to control rats. 35.6 ± 0.6% and 26.4 ± 1.5% decreased-relaxation was reported for 10^-5^ M ACh-induced EDR (Figure 2) for 10^-6^ M ADPβS-induced EDR (Figure 2), respectively.

### Effect of Intense exercise training on ACh and ADPβS- induced EDR in control and STZ-diabetic rats

**Figure 3:**
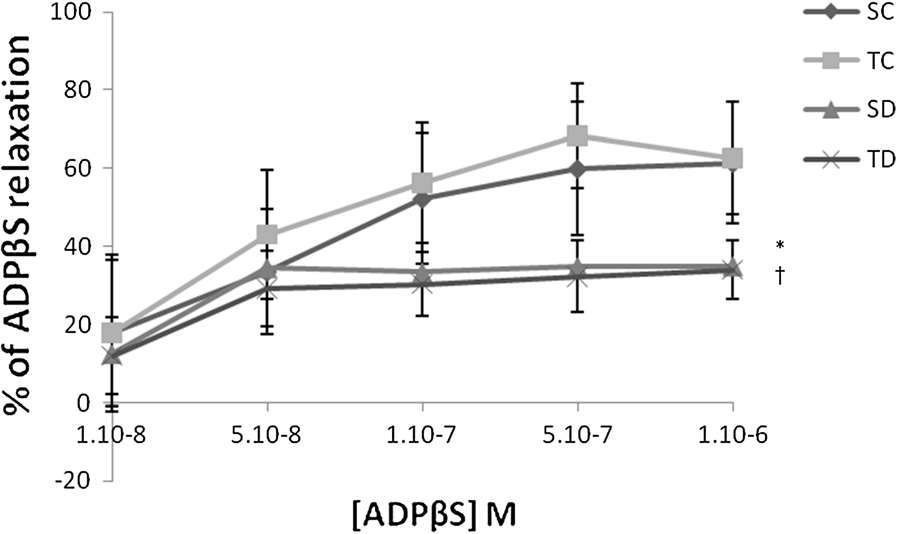
**Concentration dependent relaxation ADPβS (%).** Concentration dependent relaxation of ADPβS on thoracic aorta rings obtained from sedentary control (SC), trained control (TC), sedentary diabetic (SD) and trained diabetic (TD) groups precontracted with PE. Tension is expressed as % relaxation on initial contraction with PE. Values are expressed as mean ±SEM. * significantly different from sedentary control rats (p<0.05), † significantly different from trained control rats.

The concentration-response curves to ACh and ADPβS were not different between TC vs SC and TD vs SD rats (Figures 2 &3).

### Effect of L-NAME on ACh and ADPβS-induced EDR in control and STZ sedentary diabetic rats

**Figure 4:**
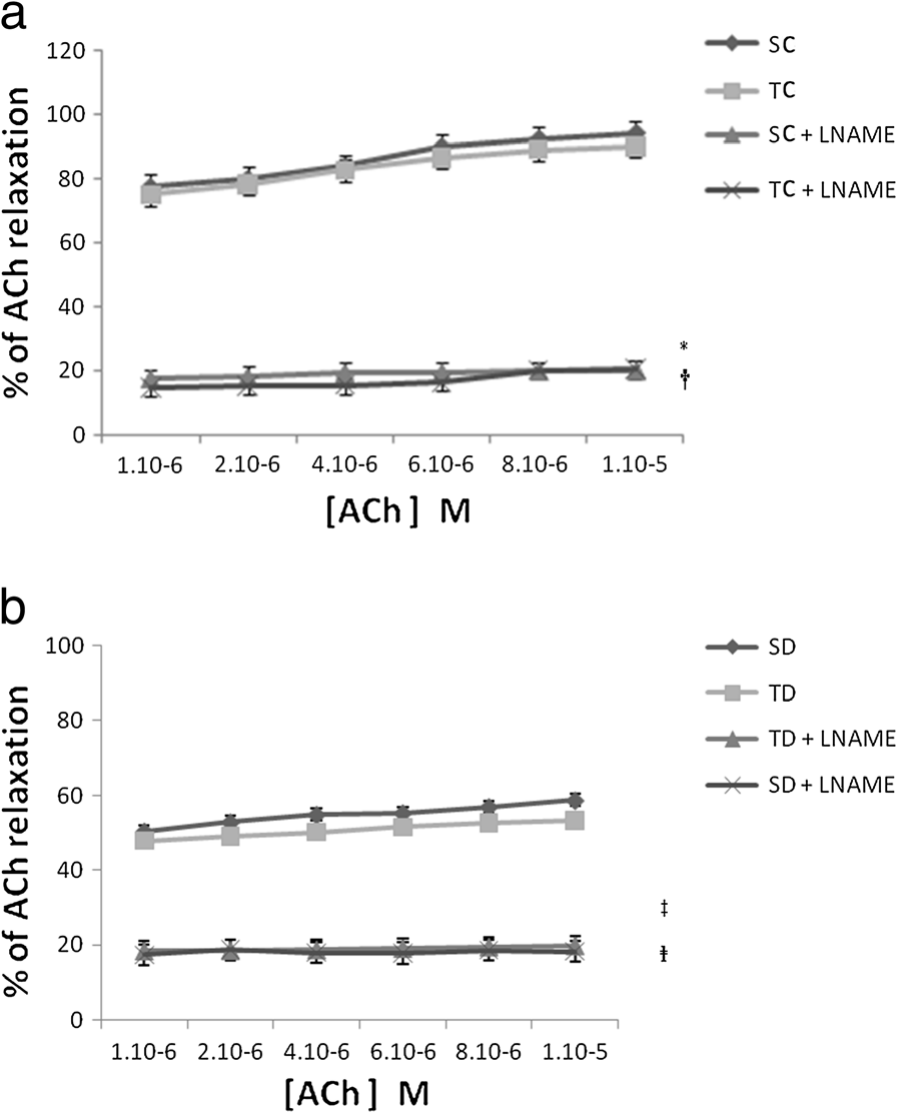
**(a) ACh-induced relaxation with and without LNAME in sedentary and trained control.** Concentration dependent relaxation of acetylcholine (ACh) with and without LNAME on thoracic aorta rings obtained from sedentary control (SC), trained control (TC) group precontracted with PE. Tension is expressed as % relaxation on initial contraction with PE. Values are expressed as mean ±SEM. * significantly different from sedentary control rats (p<0.05), † significantly different from trained control rats. **(b)** ACh-induced relaxation with and without LNAME in sedentary and trained STZ-diabetic rats. Concentration dependent relaxation of acetylcholine (ACh) with and without LNAME on thoracic aorta rings obtained from sedentary diabetic (SD) and trained diabetic (TD) groups precontracted with PE. Tension is expressed as % relaxation on initial contraction with PE. Values are expressed as mean ±SEM. ‡ Significantly different from sedentary diabetic rats (p<0.05). Significantly different from trained diabetic rats (p<0.05).

**Figure 5:**
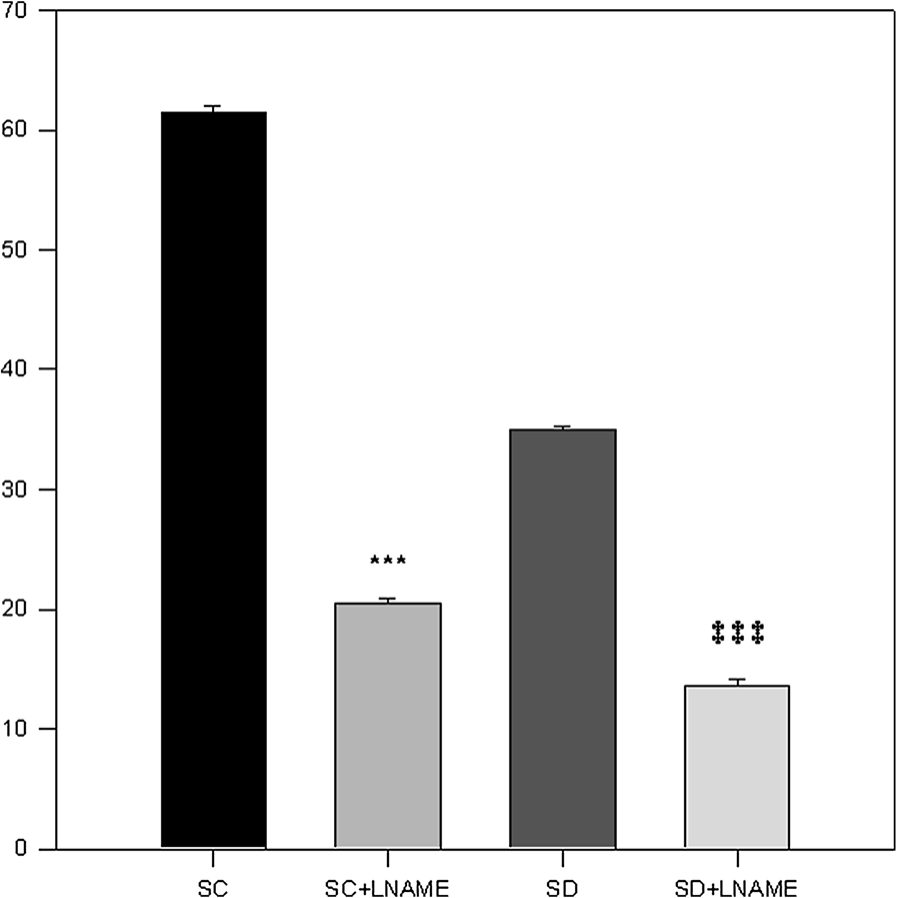
**ADPβS-induced relaxation with and without LNAME.** Concentration dependent relaxation of ADPβS with and without LNAME on thoracic aorta rings obtained from sedentary control (SC) and sedentary diabetic (SD) groups precontracted with PE. Tension is expressed as % relaxation on initial contraction with PE. Values are expressed as mean ±SEM. *** = p<0.001 significantly different from (SC). ‡ ‡ ‡ = p<0.001 significantly different from (SD), groups.

The L-NAME (10^-6^ to 10^-5^ M) inhibits the relaxations induced by ACh (Figure 4a, b) in both groups (healthy and diabetic). Less than 20% relaxation induced by Ach was still preserved. In the same way, L-NAME combined with ADPβS (10^-6^ M) limited the EDR to about 20% in both groups (Figure 5).

## Discussion

Our study showed that diabetes decrease ACh and ADPβS-induced EDR about 35% and 26% , respectively. In this population, the ADPβS-induced EDR constituted original data. Intensive endurance training did not induce beneficial effects on EDR aorta in control group and did not reverse the vascular dysfunction induced by the diabetic condition. In all groups, under L-NAME, the decrease in ACh or ADPβS-induced EDR confirmed that endothelial relaxations are mediated via NO pathway.

In our diabetic rat model, the higher blood glucose concentration compared to healthy animal one confirmed the absence of insulin secretion subsequent to the STZ-treatment. Our training program lead to the level decrease the level of blood glucose, which was lower in TD compared to the values of their counterparts. Moreover, the drastic decrease in body weight reinforced the diabetic status of animals. Training of diabetic rats led to weight gain due to the increased-muscle mass, reduced weight loss but did not restore a normal body weight such as in healthy animals. Therefore, these observations are in agreement with literature data reporting changes in diabetic (type 1) animals body weight [8,24]. The CS increase activity demonstrated the efficiency of our training program. The same training program induced a higher increase in CS activity in STZ-diabetic rats that it did in control rats (TD, +56% vs TC, +24% ) (Table 1) because it was probably more intense for diabetic rats [22,34,35].

### The vasoconstriction increase is induced by PE in STZ-diabetic rats

We observed a significantly increase in contractility induced by PE in diabetic groups as compared to the control ones for concentrations from 10^-7^ M to 6. 10^7^ M. Therefore, the different concentration-responses curves observed were in agreement with Martinez-Nieves and Dunbar study (1999) [36]. These authors postulated that changes in vascular smooth muscle could be involved in the vascular tone increase observed in diabetic subjects. The mechanism of this potentiation of the PE-induced contraction is due to increasing sensitivity of alpha adrenergic-receptors [36].

### The decrease in ACh and ADPβS-induced EDR supports an endothelial dysfunction in STZ-diabetic rats

Diabetes is known for reducing the ACh-induced relaxation [21,37,38]. The nature of the studied receptors mediating respectively ACh (Muscarinic (M1/3) receptors) and ADPβS (Purinergic (P2Y) receptors) induced EDR could have an impact on the importance of reducing the EDR in diabetic rats. ADPßS has been shown as an inducer (via P2Y purinergic receptors) of insulin secretion by the beta cells and vasodilatation of pancreatic vascular bed [39]. It is important that along with the secretion of insulin, a vasodilatation must occur. This vasodilatation fosters the effect of insulin and therefore responds to the metabolic and energetic needs. Moreover, if we take into account the physiopathology, we may notice that a dysfunction in the endothelium of pancreatic vascular bed can only perturbate the glycemia regulation. It appeared that the reduction of the EDR was higher in diabetic rats when induced by ACh than by ADPβS, respectively. ACh as ADPβS induced EDR are mediated by NO released from endothelium and consecutively the stimulation of soluble Guanylate cyclase (GMPc). The decrease in NO bioavailability was probably one of the reasons in this relaxation alteration. Therefore, the histamine-induced EDR was potentiated on the diabetic rat [40]. In diabetic rats, the decrease in ADPβS-induced EDR was supported by previous reports [41].

It is obviously noticed that the nucleotides (such as the ADP) -present in the extracellular spaces- contribute to the local regulation of the vascular diameter as well as they play a key role in pathophysiologiacal states. Concerning the ADP and its interaction with the endothelial P2Y1 receptors in a number of diabetic rats type 1 (obtained as a result of STZ treatment), it has been recently proposed that the signalization via NO will be reduced subsequently after an interaction with P2Y1 receptors [42].

In fact, any disturbance of the cellular signalization via the P2 receptors stimulated by ADPßS at the level of the endothelium of pancreatic vascular bed as well as at the level of the cells β, can only lead to a dysfunction regarding the glycemia regulation. A new trend of research appears to remove and rectify this dysfunction targetting the system of signalization mediated by purinergic receptors P2Y.

Indeed, the ADP (as well as ATP, AMP, and adenosine) can hyperpolarize the membrane of the endothelial cell. However, ATP and ADP depolarize the membrane of the smooth muscular cell [43]. As noticed before, associated with the P2Y1 (endothelial receptors) ADP or ADPßS would stimulate the production of EDHF by endothelial cells. In fact, EDHF can induce hyperpolarisation of the vascular smooth muscle membrane thus a vasodilatation would occur. ADPßS is very important for glucose regulation because Hillaire-Buys et al. (2003) [44] reported that ADPßS linked to P2Y was able to alter endothelium-dependent vasorelaxation in vascular pancreatic bed. Specifical agonists as well as ADPßS to P2Y- receptors induced NO/EDRF and EDHF secretion by endothelial cells. EDHF act on vascular smooth muscle cell, openning K^+^ channel, induce hyperpolarisation and relaxation [40].

### Intense exercise training did not potentiate and/or reverse the ACh and ADPβS-induced EDR in control and in STZ-diabetic rats

Our intensive training program did not potentiate and/or reverse the ACh and ADPβS-induced EDR in both groups. This phenomenon could be explained by an excessive ROS production as compared to the positive shear stress effect induced by this exercise. In fact, physical exercise inducing shear stress is the main stimulus of the NO pathway [45]. Shear stress stimulates the membrane receptors sensitized to the stretching of the endothelial cells, and as a result exercise induces vasodilatation. Consequently, EDR should have been greater with training. On the other hand, intense and exhaustive exercises are well known to induce oxidative stress and an excess of ROS production [46]. Previous studies realized on isolated thoracic rat aorta in hypoxic conditions (using drugs) showed that ROS (02°^-^, 0H-…) alters vasomotricity [47]. NO capturing through the superoxide anion associated with a production of pro-oxidant reactive oxygen species (ROS) can support this hypothesis. Moreover, a correct EDR via the NO°/EDRF pathway requires a coupling between receptors, NOS and cofactor BH4. The inefficiency of our exercise training program to reduce the ED (and enhance EDR) in diabetic rats can be explained by the fact that intense training promotes oxidant stress and leads to decoupling eNOS-cofactor BH4 [17,35,48]. Internal data of our lab supported this hypothesis. Indeed, diabetic rats or trained diabetic rats exhibited an hyperglycemic-induced O2°^-^ overproduction, resulting from activation of NOX, eNOS, and Xanthine Oxidase.

In addition, the production of superoxide anion, lactic acid and other molecules may be involved when the shear stress is too high**.** Consequently, we hypothesized that our endurance training was performed at too great intensity for diabetic population, while for more moderate intensities beneficial effects are typically observed on EDR [15]. Consequently, we can conclude that intense training program was not adapted to reduce an ED in type 1 diabetic rats.

### ACh and ADPβS-induced EDRs are mediated via NO pathway in control and STZ-diabetic rats

Our results demonstrated that the NO°/EDRF pathway is involved in the molecular mechanism of these EDR as described in other studies [29,31]. In particular, we observed that the NO°/EDRF pathway was implicated in the ACh and ADPβS-induced EDR at the same ratio as in control and STZ-diabetic rats. The fraction of ACh and ADPβS-induced EDR not inhibited by L-NAME could be eventually mediated by PGI2 or EDHF [31]. Consequently, it would be interesting to explore the consequences of training in normal or diabetic animals, towards prostacyclin and that of EDHF. It will be intriguing to pursue these investigations particularly with regard to molecular interactions and the metabolism of NO with molecules involved in oxidative stress (superoxide anion, hydroxyl anion, superoxide dismutase, catalase…), as demonstrated in isolated rat thoracic aorta in hypoxic conditions [38,49]. Classically, it was described that oxidative stress is increased by exercise training [50-52]. When normally at rest, however, a low oxidative stress level is maintained. Obtaining a correct EDR via the NO°/EDRF pathway requires a coupling between receptors, NOS and cofactor BH4. It would be interesting in the future to determine whether the reduction of EDR in diabetic rats is due to BH4-eNOS uncoupling or another molecule.

## Conclusion

This study showed that intense exercise training cannot correct endothelial dysfunction, as measured by ACh and ADPβS-induced EDR. EDR did not change in diabetic rats after 8 weeks of intense exercise training. Such findings will most likely recommend the practice of moderate intensities for exercise training to induce benefits on vascular function. It is necessary to pursue a better understanding of the mechanisms of ED in the future.

## Competing interests

The authors declare that they have no competing interests.

## Authors’ contributions

MSZ was responsible for the design conception of the experiments, collection, analysis and interpretation of the data, and drafting of the manuscript. SV was responsible for the conception of the experiment and collection of the data. SLDL and LM were responsible for exercise training protocol. ZT has participated in interpretation of the data. BS supervised the study and was responsible for the design conception of the experiments, interpretation of the data, and drafting of the manuscript. All authors have read and approved the final manuscript.
